# Multiscale Molecular Dynamics Simulation of Multiple Protein Adsorption on Gold Nanoparticles

**DOI:** 10.3390/ijms20143539

**Published:** 2019-07-19

**Authors:** Francesco Tavanti, Alfonso Pedone, Maria Cristina Menziani

**Affiliations:** Department of Chemical and Geological Sciences, University of Modena and Reggio Emilia, Via Campi 103, 41125, Modena, Italy

**Keywords:** multiscale computational simulations, plasma proteins, nanoparticle, competitive binding, protein corona

## Abstract

A multiscale molecular dynamics simulation study has been carried out in order to provide in-depth information on the adsorption of hemoglobin, myoglobin, and trypsin over citrate-capped AuNPs of 15 nm diameter. In particular, determinants for single proteins adsorption and simultaneous adsorption of the three types of proteins considered have been studied by Coarse-Grained and Meso-Scale molecular simulations, respectively. The results, discussed in the light of the controversial experimental data reported in the current experimental literature, have provided a detailed description of the (i) recognition process, (ii) number of proteins involved in the early stages of corona formation, (iii) protein competition for AuNP adsorption, (iv) interaction modalities between AuNP and protein binding sites, and (v) protein structural preservation and alteration.

## 1. Introduction

Gold Nanoparticles (AuNPs) have widespread prospective applications in biomedical fields, including drug delivery, imaging, hyperthermia, biosensors, and biocatalysis. For this reason, there is an urgent need to improve the still limited understanding of their short- to long-term environmental effects [1].

When the AuNPs are injected into biological systems, a number of proteins can be absorbed on their surface, forming the so-called “protein corona” [1,2,3,4]. The protein adsorption process is a dynamic and competitive phenomenon. In the early step of contact, a “soft corona” made by proteins with higher concentrations is formed, but progressively proteins with higher affinity will replace the previous adsorbed proteins, forming the “hard corona” (the Vroman effect) [5,6,7]. 

Several factors influence the composition and features of the protein corona, such as nanoparticle (NP) composition, size, shape, ligand coating, time of the exposure, nature of the physiological environment, relative abundance of NPs and proteins, and protein affinity to NPs [8,9,10]. 

It is widely acknowledged that the protein corona on the NP dramatically changes the nature of interactions between the nanoparticle and its biological environment. Adverse health effects of NPs can be ascribed to the alteration of the biomacromolecule conformation and activity, as indicated by recent clinical data [11,12].

A wide variety of experimental methods exists to gain information on thickness and density of the corona and identity, quantity, arrangement, orientation, conformation, and affinity of proteins constituting the NP corona [4]. A common challenge of both direct (mass spectrometry, circular dichroism, etc.) or indirect (via measuring modifications in the NPs properties, such as changes in size) analyses is the isolation of the nanoparticle–protein complex from excess proteins, without disrupting the complex or inducing additional protein binding. Moreover, limited arrays of in situ techniques, which present the advantage of measuring without the need of removing unbound proteins, are also available. Yet, these techniques are not straightforward to use, and great care has to be exercised in order to produce results with a high confidence level and to avoid erroneous conclusions. Moreover, robust and precise standard operating procedures are still lacking and significant variations in the results obtained by different experimental techniques or by the same experiment from different laboratories are often observed [4,13].

Therefore, our ability to understand the physical principles governing protein–NP interactions using experimental methods alone is strongly limited.

Molecular dynamics (MD) simulations provide a complementary tool for improving our understanding of the protein-AuNP interactions at the molecular level, as demonstrated by an increasing number of studies and applications appearing in the literature [14,15,16]. 

In this study, MD simulations at different level of scales have been carried out to characterize the interaction between three proteins, i.e., myoglobin (MB), hemoglobin (HB), and trypsin (TRP), and a 15 nm diameter citrates-capped AuNP. 

The proteins chosen can be considered as “model proteins”, characterized by a compact and almost spherical shape, increasing dimensions, and different number of surface exposed Cys residues myoglobin (MB): monomer, 153 amino acids, 17.9 kDa, no Cys residues; hemoglobin (HB): tetramer, 574 amino acids, 65 kDa, no exposed Cys residues; trypsin (TRP): monomer, 223 amino acids, 23KDa, 2 partially exposed Cys residues engaged in a disulfide bridge). Moreover, their three-dimensional structure is well established and comprises different secondary structures arrangements (α-helices and β-sheets). Finally, a number of experimental findings is available in the literature reporting on these proteins interacting with citrates-capped AuNPs of various dimensions [17,18,19,20,21,22,23,24].

In this paper, the driving forces for bio-corona formation, protein binding sites, protein conformational changes, AuNP adsorption capacity, and protein competition for AuNP binding will be discussed, and the results will be compared with the available experimental findings, providing hints for the resolution of the controversial picture that may emerge by comparing data from different experimental laboratories.

## 2. Results and Discussion

### 2.1. Driving Forces for Bio-Corona Formation 

The driving forces for adsorption of proteins on the AuNP surfaces can be identified by the analysis of the amino acid residues that approach the AuNP surfaces in the early stage of the coarse-grained (CG)simulation time. In the CG simulation a contact is established when the distance between the amino acid α-carbon of the protein and the NP surface is smaller than 6.5 Å.

**Figure 1:**
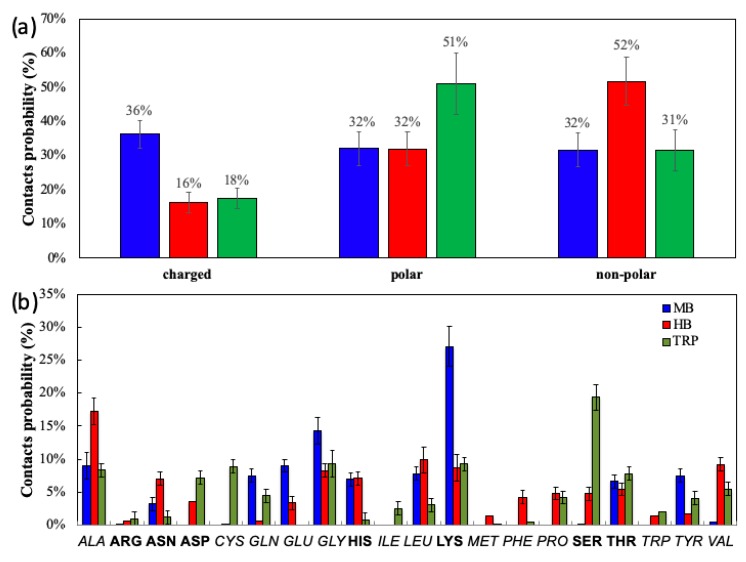
(**a**) The contact probability of the amino acids of MB (blue), HB (red) and TRP (green), clustered in non-polar, polar, and charged regions; (**b**) the contact probability of each amino acid of the three proteins; the non-polar amino acids are reported in italic, the polar and charged amino acids in bold. Statistic difference in the bar chart are reported.

Figure 1 shows the contact probability for each amino acid residue of MB, HB, and TRP interacting with the AuNP. The electrostatic interactions between charged and polar amino-acids and the AuNP drives the adsorption of the MB proteins (68% of the total contacts), as previously speculated by Sevilla et al. [17] on the basis of the results of their experimental investigation, in which they studied the concentration-dependent interaction between MB and citrate-capped gold nanospheres. In particular, Lys amino acids make the highest number of interactions with the AuNP, possibly attracted by the metal-adsorbed citrates.

On the contrary, the binding of HB and AuNP is mainly driven by Van der Waals forces (52% of the total interacting residues are non-polar), as previously experimentally found [18,19]. This is confirmed by the high number of contacts that non-polar amino acids make with the AuNP, and in particular Ala, Gly, Leu, and Val amino acids.

The driving force for TRP adsorption onto AuNP is given by polar amino acids (51% of the total interacting residues are polar), of which Ser shows the highest number of contacts (Figure 1). 

### 2.2. Protein Binding Sites

The modalities of adsorption of the proteins onto the NP have been analyzed further by tracking the protein regions that contact the NP surface and produce persistent interactions during the course of the computational simulations. An interaction is defined as persistent, and therefore constituting the protein binding site if the amino acid residue remains in contact with the metal for at least 70% of the total protein-NP surface interaction time [25]. The amino acid residues that give persistent interactions and whose lateral chains are exposed to the NP surface are reported in bold in the following discussion.

**Figure 2:**
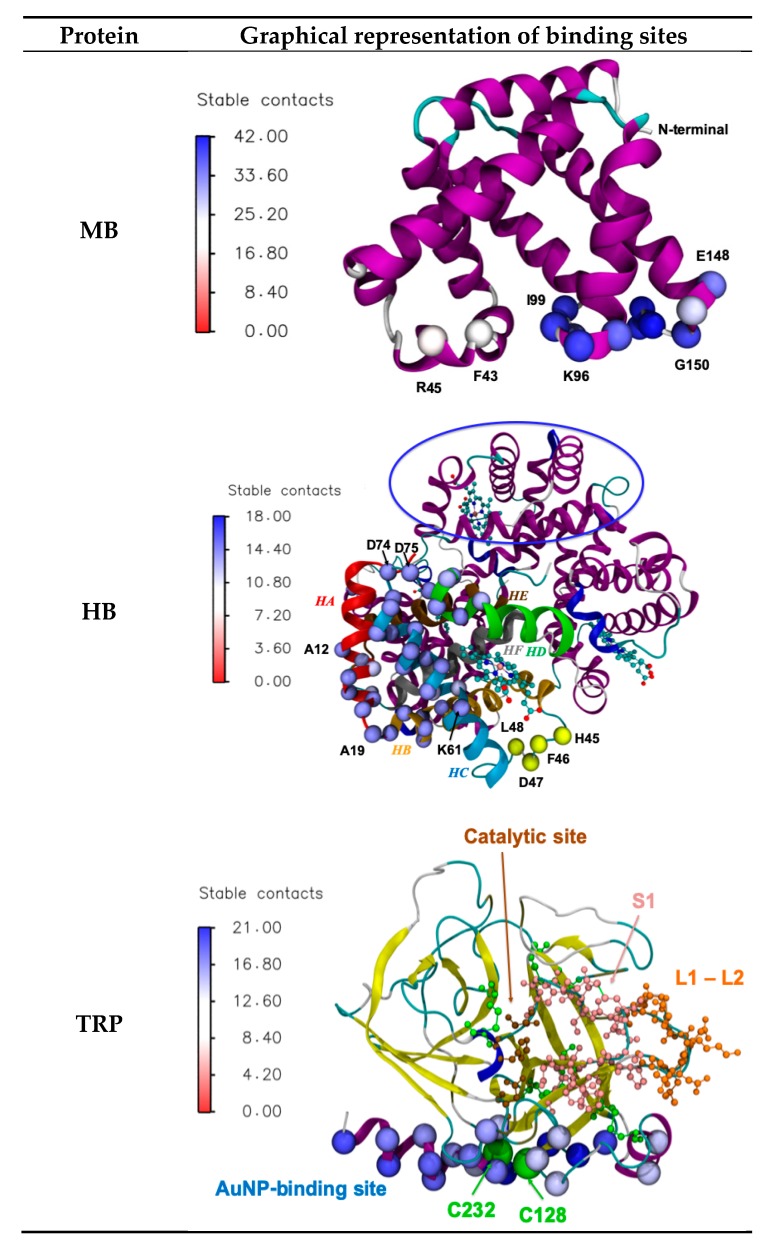
Secondary structure representation of the three proteins studied; spheres represent the α-carbon of amino acids in contact with the AuNP, colored accordingly to the number of stable contacts, as shown in the legend. For clarity’s sake, the amino acids of HB in contact with the AuNP surface are shown in the first sub-unit only, whereas the analogous contact region in the second sub-unit is highlighted by a blue circle. Helices of chain A are colored in red (Helix A), orange (Helix B), light blue (Helix C), green (Helix D), brown (Helix E), and grey (Helix F). The second binding site in the loop between helices C and D is shown in yellow. For TRP, the cysteine residues are represented in green and labelled with their sequence number; the location of the catalytic site and of the primary substrate-binding pocket is highlighted.

The results of CG simulation show that the binding site of MB towards the AuNP is very specific; it is located at the C-terminal region of the protein and encloses residues belonging to the stretches of amino acids ^96^KHKI^99^, and ^146^YKELGYQG^153^; the F43 and R45 residues also contribute to the binding (Figure 2). This binding site hypothesis is consistent with the results of the surface enhanced Raman spectroscopy (SERS) study on the concentration-controlled formation of horse muscle MB/Gold nanosphere aggregates carried out by Sevilla et al. [17]. In fact, the observed SERS spectrum reveals the involvement of the C-terminal part of the sequence, corresponding to the G- and H-helices, where the only two tyrosine residues of the horse MB (residues 103 and 146) are situated, whereas the bands of the tryptophan residues lying close to the N-terminal are silent in the SERS spectrum. In the Physeter catodon MB used in this study, Y103 is not directly bound to the AuNP surfaces but lays on the side of the protein surface buried by the NP; the Physeter catodon MB presents an additional Y at position 151 (F in horse muscle MB considered in this study) that contributes to the binding. 

In the case of HB, two main regions of chains A and C interact with the AuNP, as shown in Figure 2. The stretches of amino acids involved are: ^12^AAWGKVGAHAGEYG^25^ in helices HA and HB; ^61^KVADALTNAVAHVDDMPNALS^81^ in helices HC and HD. Moreover, ^51^PDA^53^ of chain B and ^45^HFDLSPDA^53^ of chain D constitute the binding regions of proteins approaching the already crowded AuNP surface. In fact, the interactions with the surrounding proteins force the contact between HB and the surface through the protruding loop of the protein connecting helix B and C.

Analysis of the thermodynamic parameters carried out for the interaction of human hemoglobin/18-20 nm gold NP and horse hemoglobin/25 nm gold NPs [17,18] indicates that the interaction is spontaneous and is mainly dominated by hydrogen bonding through Tyr residues and van der Waals interactions. Moreover, as for MB, a direct interaction with Trp residues is ruled out [18]. In our study, neither Y24 nor W14, although laying in the region involved in the NP interaction, are in direct contact with the NP surface.

The binding region of TRP towards the AuNP is located at the C-terminal α-helix (^231^VCNYVSWIKQTS^244^) and is extended to a number of spatial surrounding amino acids: Y94, ^125^TSCASAGTQ^135^, S166, S167 (Figure 2). This zone of the protein contains the disulfide bridge composed by C128 and C232; these are the only Cys residues located on the TRP’s surface that could in principle make contact with the NP, whereas the Cys engaged in the other 5 disulfide bridges are located in buried or core regions. In this study, the Cys-Au covalent interactions are not considered, since experimental evidence [26] has shown that appropriate stoichiometries of the Trp–AuNP construct preserve its secondary structure and is more resistant to autolysis and chemical denaturation as compared to the native trypsin (Section 3.3). On these bases, Nidhin et al. [21] hypothesized that the TRP/AuNP interaction is established by fast electrostatic and dispersive forces, and that no slow formation of covalent bond between the exposed C residues and the AuNP is likely to occur. Electrostatic and hydrophobic interactions were also found to be mainly responsible for binding between TRP and 13 nm AuNPs by Zhang et al. [23] by means of an experimental multi-technique approach. On the basis of the results obtained by docking modeling exercises, these authors claim that the AuNPs binding site is located near to the primary substrate-binding pocket and the active site of the enzyme substrate. In our study, the AuNP binding site does not hinder the catalytic site of TRP (H57, D102, and S195), the primary substrate-binding pocket called S1, represented in pink in Figure 2 (^189^DSCQGDS^195^, ^214^SWGSGC^220^, and ^225^PGVY^228^) and the two loops near the S1 site, called L1 and L2, represented in brown in Figure 2 (^185^LEGG^188^ and ^221^AQKN^224^).

Discrepancies in the computational findings can be due to the fact that Zhang et al. [23] used for their docking calculation an Au32 icosahedral nanocage, whose dimension (~0.85nm in diameter) and surface properties were very different from the AuNPs used in the present study.

### 2.3. Proteins Conformational Changes

It is widely acknowledged that in controlled experiments where all other conditions are held constant, the effect of secondary structure disturbance upon protein interaction with metal surfaces strongly depends on the ratio protein/NP size and on NP surface hydrophobicity [27]. In particular, proteins are generally more stable on small NP with a hydrophilic surface.

All the experimental studies reported in the literature on the interaction between AuNP and MB, HB, and TRP proteins [17,18,19,20,21,22,23,24] make use of citrate-coated NP, but are characterized by the use of different experimental techniques, different operating procedures, and a wide range of NPs of different sizes, from 12 nm to 70 nm. Therefore, the comparison among the results is difficult and highlights contradictory interpretations. In this scenario, although in a qualitative way, the computational results obtained in this work on the variation in the secondary structure of the MB, HB, and TRP upon NP adsorption can provide a rationalization of the available experimental evidences. Table 1 lists the percentage of secondary structure elements observed from the CG simulations in the native proteins and upon adsorption on two NPs of diameter 15 and 70nm. The first NP shows an approximate protein/NP diameter ratio of 1:3 and can be considered a small NP, while the second is 1:15 and can be considered a medium NP with respect to the dimension of the proteins studied. 

The secondary structure of MB after adsorption over the 15 and 70 nm AuNP shows a small decrease in the helical content of the order of 5% to 10%, respectively, with a consequent increase of the unordered structures. A significant loss in a is α-helix content (not quantified in the paper) is observed by circular dichroism and surface enhanced Raman spectroscopy [17] for MB bound to citrate-capped 30 nm gold nanospheres, although the authors stated that the amount of observed chemical denaturation strongly depends on the protein concentration used and is never so drastic as to lead to heme loss. On the contrary, no changes are observed by FT-IR spectroscopy [22] in the secondary structural composition of MB bounded to a 40nm AuNP as a function of incubation time. 

The decrease in the helix content of HB upon NP binding of both 15 and 70 nm is in the order of the 5%. This result nicely agrees with the 3% to 6% changes in the helix content of HB adsorbed over 15nm AuNP observed by Chakraborty et al. [18] by means of Circular Dichroism. Moreover, UV-vis and fluorescence analysis [19] showed that horse HB retains its native structure after the binding with AuNP.

These results are of major importance in order to support the HB/AuNP system as components of electrochemical sensors, biosensors, and drug delivery.

Although no loss in helix content is observed upon TRP binding to AuNP, 9% of β-sheet increase characterizes the binding to the big 70nm AuNP. (Table 1). 

Recently, Nidhin et al. [21] found that the stoichiometry of 12–14 nm AuNP-protein constructs is a strategic factor that influences structural and functional losses either in protein or nanoparticles. In fact, circular dichroism spectra show that the conformation of 1:1 TRP/AuNP complex is almost identical to that of native trypsin, whereas changing the stoichiometry in favor of the NPs results in a significant loss of β-sheet conformation.

Wang et al. [22] observed, through FT-IR spectroscopy, significant changes in the secondary structural composition of TRP-AuNPs by using NP of 40 nm of diameter. The authors ascribe the changes in protein structure over incubation time to a fast absorption dominated by electrostatic forces, followed by slow formation of a S-Au covalent bond with Sulphur in disulfide bridges, which being located in flexible buried regions takes time to come into contact with NPs. It is worth noting that the molecular simulations carried out in this study considers a classical description of the system and are suitable to describe only the early stages of the process, the variation in secondary structure due to surface interaction, but not the formation of the S-Au covalent bond. 

### 2.4. Protein Adsorption Capacity of Gold Nanoparticle

The characterization of protein binding to NP (protein binding capacity, thermodynamic parameters, etc.) by means of several experimental techniques relies on the esteemed number of proteins adsorbed on a single AuNP [13]. Usually, simple geometric considerations are used by experimentalists. However, as is shown in Table 2, the results obtained strongly depend on the approximations used. 

For example, the method proposed by Wang et al. [28] is based on the assumption that proteins are compact entities that can be described by their radius of gyration, which do not change upon adsorption. The methods of Calzolai et al. [29] assumes a very close packing of the proteins over the AuNP; the crude approximation of the protein shape projected as a flat circle onto the NP surface is the basis of the method used by Dell’Orco et al. [30]. None of these methods consider the affinity of the proteins to the AuNP that resides in the protein shape and surface characteristics. 

Although the maximum number of proteins that can be bounded to AuNP cannot be obtained directly from the coarse-grained simulations due to the high computational cost for long-time simulations with large numbers of proteins, it can be estimated by fitting the trend of the variation of the number of bound proteins (*N_bound_*) with time by means of a stretched exponential function [31]:

(1) $$N_{bound}=N_{max}\left(1-e^{{\left(\frac{t}{\tau }\right)}^{\alpha }}\right)$$

where *N_max_* is the maximum number of proteins that can be adsorbed on the NP, *t* is the simulation time, *α* controls the width of the corresponding rate distribution, and *τ* is the characteristic time constant.

The analysis of the results reported in Table 2 show that the approaches of Wang et al. [28] and of Calzolai et al. [29] over-estimate the number of both MB and HB with respect to the computational approach, while for TRP the values computed with these two methods and with the computational approach are similar. Conversely, the equation proposed by Dell’Orco et al. [30] underestimates N_max_ for both MB and TRP. Overall, the NP surface occluded by the proteins is different from what each expected from simple geometrical considerations, where electrostatic and steric repulsion among proteins are not considered.

Recently, Zhang et al. [20] estimated N_max_ of a number of plasma proteins comprising MB, HB, and TRP, bounded to 40 and 70 nm AuNPs by means of dynamic light scattering measurements. The stoichiometry of protein-NP interactions, estimated by the hydrated diameters of proteins, NPs, and protein-NP complexes, showed a multilayer protein adsorption for all proteins. This is in contrast with the results of the CG simulations showing that, notwithstanding the excess of proteins in the simulation box, the proteins studied have a limited tendency for aggregation and the corona is composed of a single layer. The discrepancy might be explained by the protein concentration used by Zhang et al. in their experiments. In fact, according to Sevilla et al. [17], who characterized the interactions of AuNS with MB in a wide protein concentration range, i.e., from 3.7 × 10^−8^ to 7.4 × 10^−6^ M, AuNP of 30 nm diameter should be completely covered at 7 × 10^−8^ M·MB. Above this concentration, the protein molecules should either form additional layers or stay in solution. 

### 2.5. Meso-Scale Simulations of Protein Competition for AuNP Binding

In order to test the competitive adsorption between different kinds of proteins, meso-scale simulations were carried out on solutions containing the three proteins. The results are listed in Table 3.

The maximum number of proteins adsorbed on the NP surface for the three-component protein simulation is around 50 and it is reached after 15 μs. Adsorbed proteins do not show a specific pattern, but they are randomly distributed on the AuNP surface.

By comparing the number of bound proteins for one-component and three-component models, it is evident that competitive binding notably reduces the binding probability of each kind of protein, especially in the cases of HB and TRP, where the final number of adsorbed proteins decreases by a factor of around 4. Differences in the diffusion coefficients of light and heavy molecules can explain the results obtained for MB (MW 16.7 kDa) with respect to HB (MW 64.5 kDa). As for MB and TRP (MW 23.3 kDa), which are of comparable weight, analysis of the computational simulations (inset in Table 3) shows that in the first 17μs, the number of adsorbed proteins increases with a similar slope. In the final part of the computational simulations, i.e., after 17 μs, the velocity of adsorption diminishes. The local concentration of the proteins reduces the available free AuNP surface space for the binding of other proteins. At this stage, up to the end of the simulation time monitored, the total number of adsorbed proteins remains almost stable. However, stability is achieved by a consistent contribution of additional MB proteins adsorbed, at the expenses of the other proteins. Selectivity in the binding might be ascribed to the amount of Lys residues at the MB surface that give rise to strong electrostatic interaction with respect to the short-range polar forces, mainly due to surface Ser residues in TRP (Section 2.1).

Therefore, the higher concentration of MBs adsorbed on the AuNP surface in competitive studies is favored by both the diffusion coefficient and surface distribution of charged residues. 

## 3. Methods

### 3.1. Molecular Dynamics Simulations

The structures of the three proteins were retrieved from the Protein Data Bank (PDB) [32], PDB IDs: 1MBN [33] for MB, 2HHB [34] for HB, and 2PTN [35] for TRP. The electrostatic potentials mapped on the protein surfaces were computed using the APBS package [36]. Molecular dynamics simulations were performed using the DL_POLY_2.20 package [37].

### 3.2. Coarse-Grained (CG) Simulations

A coarse-grained approach, where each amino acid is replaced by a single bead located on the α-carbon, was used in order to reduce the high computational cost given by the considerable number of proteins considered in the simulation box, and to maintain a good spatial and temporal resolution [38,39,40,41]. A brief description of the Force Field [31,42] used is reported in the following:

(2) $$U=\sum^{}U_{bonded}+\sum^{}U_{non-bonded}$$

where

(3) $$\sum^{}U_{bonded}=\underset{i,j}{\sum }U_{bonds}\left(i,j\right)+\underset{i,j,k}{\sum }U_{angles}\left(i,j,k\right)+\underset{i,j,k,l}{\sum }U_{dihedrals}\left(i,j,k,l\right)$$

(4) $$\sum^{}U_{non-bonded}=\underset{i,j}{\sum }U_{non-bonded}^{local}\left(i,j\right)+\underset{i,j}{\sum }U_{non-bonded}^{non-local}\left(i,j\right)$$

The functional form of the force field comprises a short-term potential (*U_bonded_*) and a long-term potential (*U_non-bonded_*). The first term describes bonds, angles, and dihedrals of proteins. Parameters related to these potentials (*R*, *θ_0_* and *φ_0_*) are calculated on the reference structure of proteins. Force field parameters for proteins are reported in previous work [39,40,41]. Non-local, non-bonded interactions describe Van der Waals and electrostatic potentials established between the AuNP and the protein and among protein beads. Protein beads that are closer to the cutoff of 8.5 Å interact through non-bonded local terms, while beads beyond the cutoff interact with non-local non-bonded terms. The cutoff for the non-local non-bonded terms is set to 12 Å [38].

For protein beads, VdW interactions, i.e., excluded volume interactions between two beads distant *r_ij_*, were described by a Morse potential:

(5) $$U_{VdW}=\varepsilon \left[{\left\{1-e^{-\alpha \left(r_{ij}-r_{0}\right)}\right\}}^{2}-1\right]$$

with ε=0.01 kcal/mol, α = 0.70 and r_0_ = 0.95nm [38].

The protein-AuNP interactions were modeled with a Lennard-Jones potential. The interaction parameters were derived from the work of Nawrocki and Cieplak [43] that obtained the binding energy, *ε*, and the bond length, *σ*, for amino-acids interacting with gold using three different force fields. As they stated, the Force Field by Bizzarri et al. [44,45] (called FFB) is the one with the highest sensitivity for amino acids and it gives best results when no cysteine amino acids are involved. The interaction energy has been scaled by a factor of 13, which represents the average number of gold atoms interacting with each amino acid at the atomistic scale, obtained by considering an average size of particle surface area (Au {100} and a {111} surfaces) for amino acids of 0.4 nm^2^.

Water was implicitly treated in the model using a dielectric constant in order to greatly decrease the computational cost [31,42]. Electrostatic interactions between two beads of charge *z_i_* and *z_j_* at a distance *r_ij_* are given by the Debye-Hückel potential:

(6) $$U_{elec}=\underset{i,j}{\sum }\frac{z_{i}z_{j}e^{2}}{4\pi \varepsilon_{0}r_{ij}}e^{-\frac{r_{ij}}{l_{D}}}$$

where *l_D_* is the Debye length, defined as $l_{D}={\left(8\pi l_{b}I\right)}^{-\frac{1}{2}}$, and *l_b_* is the Bjerrum length at room temperature, which is set to 7 Å. I is the ionic strength, corresponding to an ion concentration of 150mM, and the dielectric constant, *ε*, is set to 10, as was done previously [31,42].

A single gold nanoparticle with a diameter of 15nm was considered. It was built as a hollow sphere made of 5160 gold beads. To each bead was assigned a charge of –0.5 (e) in order to simulate the presence of a uniform coating of a single layer of citrates over the AuNP surface [46]. By considering the NP size, about 860 citrates are estimated to be present on the AuNP surface. 

The cubic simulation box, whose side was set to 75 nm, contained one AuNP and 100 protein replicas of each protein type with a random initial position.

The system was first equilibrated at increasing temperatures ranging from 10 to 310 K with steps of 50 K, where the temperature was controlled by a Berendsen thermostat with a coupling time of 10 fs. Then, three independent 500 ns production runs at 310 K were performed by changing the starting velocities and using a stochastic thermostat with a coupling time of 10 fs and a timestep of 10 fs. 

Protein conformational changes upon binding 70 nm AuNP were detected by means of CG simulations of singe proteins docked to the NP surface in the preferred conformations observed in the previous simulations and in the same operational conditions.

### 3.3. Meso-Scale (MS) Simulations

**Figure 3:**
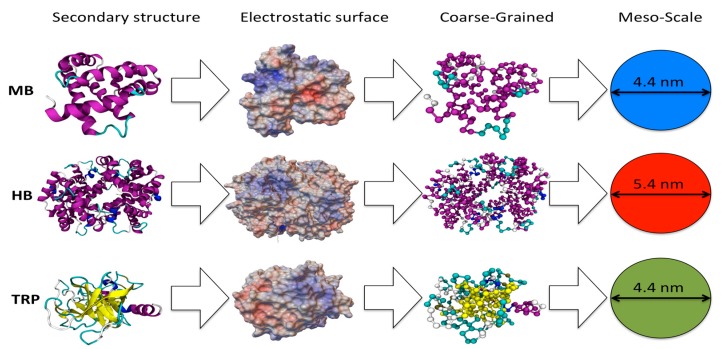
Models of the myoglobin (MB), hemoglobin (HB), and trypsin (TRP) proteins at different scale-levels. From the left, the secondary structure representation of the studied proteins colored according to their secondary structures, protein electrostatic potential surfaces, the Coarse-Grained model, and the Meso-Scale representation.

The competition between proteins during the formation of the bio-corona in the early steps was studied by means of the meso-scale approach previously used for the adsorption onto surfaces [26] and on pristine nanoparticles [47]. In this model, each globular protein is treated as a single sphere with the same mass (*M_i_*) and radii (*R_i_*) of the real protein (see Figure 3), and the interactions between proteins and NPs are treated using the DLVO theory [48,49]. The interactions between two proteins (*i* and *j*) are described by the functional form

(7) $$U_{prot_{i}-prot_{j}}=\varepsilon_{ij}{\left(\frac{\sigma_{ij}}{r}\right)}^{24}$$

where r is the distance between proteins, ε_ij_ is the interaction strength, and σ_ij_ is the sum of the two protein radii. The interactions between proteins are considered negligible with respect to the interactions between proteins and NP [26]. This assumption is protein-dependent [31,42]; in this work, the approximation is justified by the results of the CG simulations carried out in the previous step, where no aggregation of proteins was observed in the time space studied. 

The interactions between proteins and NPs are described by a 24–12 potential

(8) $$U_{NP-prot_{i}}=4\varepsilon_{NP-prot_{i}}\left[{\left(\frac{\sigma_{A}}{z}\right)}^{24}-{\left(\frac{\sigma_{A}}{z}\right)}^{12}\right]$$

where z represents the distance between the centers of mass of the NP and the protein, ε_NP-prot_ is the interaction strength of proteins with the NPs obtained by the experimental association constant (*K_a_*), and

(9) σA=Ri2112

to consider the possible distortion of the protein structure after binding.

The system modelled consists of one spherical NP of 15 nm diameter surrounded by 2400 spheres, each representing one protein placed in a random position. The ε_NP-prot_ interaction parameters were derived by making use of the association constant (*K_a_*) data values measured for the proteins of interest by Zhang et al. [20], and refining them in order to reproduce the number of adsorbed proteins of the CG simulations (MB *K_a_* = 6.6 × 10^5^ M^–1^; HB *K_a_* = 0.3 × 10^5^ M^–1^; TRP *K_a_* = 3.6 × 10^5^ M^–1^), according to the following equation:

(10) $$\varepsilon_{NP-prot}=-RTln\left(K_{a}\right)$$

The resulting ε_NP-prot_ data values ere –8.90, –7.30, and –8.59 kcal/mol for MB, HB, and TRP, respectively.

The results were refined in order to reproduce the same number of adsorbed proteins obtained by the CG model.

The systems were simulated for 25 µs with a timestep of 0.1 ps. For each protein, three independent simulations were carried out to improve the statistic.

## 4. Conclusions 

Meso-scale and coarse-grained molecular dynamics simulations have been carried out in order to improve our understanding of the protein corona formation around AuNPs. In particular, citrate-capped AuNP and three model proteins, MB, HB, and TRP, have been considered. The results provide evidence of different driving forces for bio-corona formation, mainly polar for MB and TRP and apolar for HB. Moreover, specific binding sites located at the C-terminal regions are found for MB and TRP, whereas a more extended protein surface is involved in the HB/AuNP interaction. In addition, for this last protein different binding modalities are observed depending on the NP surface occupancy. Different stoichiometries characterized the interactions of one-component and three-component models of plasma. The competition between proteins during the adsorption process affects the final composition of the AuNP protein corona and can be easily studied using Meso-Scale models.

Importantly, no significant conformational changes were observed for the three proteins in the conditions analyzed and the NP binding site did not interfere with important functional sites. Therefore, the bioactivity of the proteins was preserved and possible unexpected functions of concern for nanobiology and nanomedicine can be ruled out.

## Figures and Tables

**Table 1:** Secondary structure content for each protein before and after the adsorption over the AuNPs of 15 and 70 nm of diameter.

System	α-helix (%)		β-sheet (%)		Turn (%)		Unordered (%)	
	Before	After	Before	After	Before	After	Before	After
MB-NP15	76	71	0	0	1	1	23	28
MB-NP70		66		2		1		31
HB-NP15	76	71	0	0	2	1	22	28
MB-NP70		71		4		0		25
TRP-NP15	12	12	35	34	7	7	46	47
TRP_MP70		12		26		8		54

**Table 2:** Maximum number (N_max_) of proteins adsorbed over the AuNP calculated by means of Coarse-Grained simulations (CG) and equations provided by Wang et al. [28], by Calzolai et al. [29] and by Dell’Orco et al. [30].

Model	Protein		
	MB	HB	TRP
Wang et al. [28] *	84	35	76
Calzolai et al. [29] †	80	52	75
Dell’Orco et al. [30] ‡	25	10	22
This work CG (equation 1)	46 ± 5	18 ± 2	63 ± 5

* $N^{max}=\frac{4R_{AuNP}^{2}}{R_{G}^{2}}$, where N_max_ is the Maximum Number of Proteins, RG is the radius of gyration of the proteins (R_G_(MB) = 16.39, R_G_(HB) = 25.30, R_G_(TRP) = 17.19), R_AuNP_ is the radius of the nanoparticle determined experimental measurements. † $\;N_{max}=\frac{0.65\left[{\left(R_{AuNP}+2R_{Prot}\right)}^{3}-R_{AuNP}^{3}\right]}{R_{Prot}^{3}},\;$ where *(R_AuNP_ + 2R_Prot_)* is the radius of the AuNP-protein corona complex, *R*_AuNP_ is the radius of AuNP, and *R*_Prot_ is the radius of the protein. ‡$\;N_{max}=4\;\times \;\pi \frac{\left[{\left(R_{AuNP}-R_{Prot}\right)}^{2}\right]}{\pi \cdot R_{Prot}^{2}}$.

**Table 3:** Maximum number (N_max_) of proteins adsorbed over the AuNP calculated by means of Meso-Scale simulations (MS). On the right is the number of adsorbed proteins during a MS simulation, and on the top panel is the final configuration of the simulation.

**Protein**	**Single Protein**	**Multi Proteins**	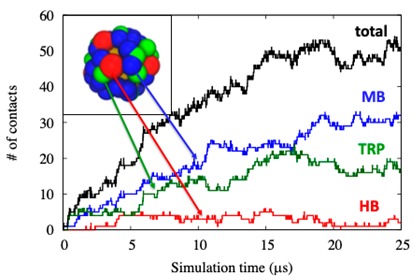
MB	44 ± 3	27 ± 3	
HB	18 ± 4	4 ± 2	
TRP	60 ± 4	17 ± 4	
